# Homologous acetone carboxylases select Fe(II) or Mn(II) as the catalytic cofactor

**DOI:** 10.1128/mbio.02987-23

**Published:** 2023-12-21

**Authors:** Krista A. Shisler, William M. Kincannon, Jenna R. Mattice, James Larson, Adam Valaydon-Pillay, Florence Mus, Tamara Flusche, Arnab Kumar Nath, Sebastian A. Stoian, Simone Raugei, Brian Bothner, Jennifer L. DuBois, John W. Peters

**Affiliations:** 1 Institute of Biological Chemistry, Washington State University, Pullman, Washington, USA; 2 Department of Chemistry and Biochemistry, Montana State University, Bozeman, Montana, USA; 3 Department of Chemistry, University of Idaho, Moscow, Idaho, USA; 4 Department of Chemistry and Biochemistry, University of Oklahoma, Norman, Oklahoma, USA; 5 Physical Sciences Division, Pacific Northwest National Laboratory, Richland, Washington, USA; University of Washington School of Medicine, Seattle, Washington, USA

**Keywords:** carboxylase, acetone, manganese, iron, CO_2_ fixation

## Abstract

Acetone carboxylases (ACs) catalyze the metal- and ATP-dependent conversion of acetone and bicarbonate to form acetoacetate. Interestingly, two homologous ACs that have been biochemically characterized have been reported to have different metal complements, implicating different metal dependencies in catalysis. ACs from proteobacteria *Xanthobacter autotrophicus* and *Aromatoleum aromaticum* share 68% sequence identity but have been proposed to have different catalytic metals. In this work, the two ACs were expressed under the same conditions in *Escherichia coli* and were subjected to parallel chelation and reconstitution experiments with Mn(II) or Fe(II). Electron paramagnetic and Mössbauer spectroscopies identified signatures, respectively, of Mn(II) or Fe(II) bound at the active site. These experiments showed that the respective ACs, without the assistance of chaperones, second metal sites, or post-translational modifications facilitate correct metal incorporation, and despite the expected thermodynamic preference for Fe(II), each preferred a distinct metal. Catalysis was likewise associated uniquely with the cognate metal, though either could potentially serve the proposed Lewis acidic role. Subtle differences in the protein structure are implicated in serving as a selectivity filter for Mn(II) or Fe(II).

**IMPORTANCE:**

The Irving-Williams series refers to the predicted stabilities of transition metal complexes where the observed general stability for divalent first-row transition metal complexes increase across the row. Acetone carboxylases (ACs) use a coordinated divalent metal at their active site in the catalytic conversion of bicarbonate and acetone to form acetoacetate. Highly homologous ACs discriminate among different divalent metals at their active sites such that variations of the enzyme prefer Mn(II) over Fe(II), defying Irving-Williams-predicted behavior. Defining the determinants that promote metal discrimination within the first-row transition metals is of broad fundamental importance in understanding metal-mediated catalysis and metal catalyst design.

## INTRODUCTION

Metalloenzymes are powerful catalysts at the junction of the geosphere and biome (1–3). Biological systems recognize, select, and construct enzymatic active sites using only the correct inorganic elements from their environments, which for bacteria are often diverse (4–7). Metal discrimination is especially challenging when Mn(II) must be selected over Fe(II). The two are adjacent in the first-row transition series and similar in size, electronegativity, and Lewis acidity. Consistent with assignment as borderline hard-soft acids, both Mn(II) and Fe(II) are found naturally in oxide and sulfide minerals (8, 9). Mn(II) is at the weak extreme of the Irving-Williams series (10–12), which orders the divalent hexaquo metals according to the exchangeability of H_2_O for an incoming ligand. This ordering suggests that thermodynamic affinity will generally oppose recruiting Mn(II), which associates weakly with incoming ligands, stably into an enzymatic binding site.

Acetone is a common organic compound used in both commercial products and industrial solvents as well as a biological product of ketone body breakdown in mammals and metabolic fermentation in some anaerobic bacteria (13). Despite its toxicity, acetone is utilized as a primary carbon source in bacteria through the activity of the metalloenzyme acetone carboxylase (AC) (14). AC catalyzes the ATP-dependent reaction of bicarbonate with acetone to produce acetoacetate, which is subsequently converted into acetyl-CoA, a central metabolite (14, 15).

ACs are complex six subunit (αβγ)_2_ bacterial enzymes that have been reported to use either Mn(II) or Fe(II) as the divalent active site metal cofactor (16–19). ACs from *Xanthobacter autotrophicus* and *Rhodobacter capsulatus* (α-proteobacteria) both contained ~1.5 mol Mn, varying amounts of Fe, and ~1 mol of Zn per (αβγ)_2_ (19–22). Physiological and biochemical studies showed that AC from *R. capsulatus* is Mn-dependent (23). In this work, growth on increasing concentrations of Mn stimulated the activity of AC in cells and the presence of Mn could be detected in cells by electron paramagnetic resonance (EPR). Further EPR studies revealed a Mn(II) signal that intensified in the presence of added nucleotide. A mechanism was proposed in which ATP coordinated Mn(II) and successively transferred its γ and β phosphoryl groups to acetone and bicarbonate. The activated carbon substrates would then combine at the same Mn(II) center, releasing AMP and two equivalents of inorganic phosphate (24).

**Fig 1:**
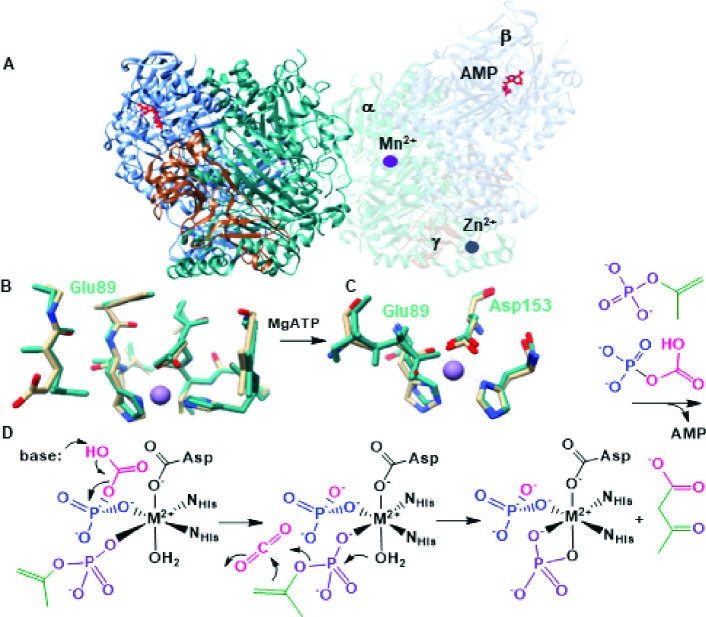
Structure and reaction of AC. (**A**) Crystal structure of *X. autotrophicus* AC with AMP (red) bound. The α subunit, containing the Mn(II) active site, is colored in teal, the β subunit with the nucleotide-binding site is colored in blue, and the Zn(II) containing β subunit is colored in wheat. One set of αβγ subunits is transparent to show the Mn, AMP, and Zn bound. (**B**) Comparison of the active sites of *X. autotrophicus* AC (teal carbons) and the *Aromatoleum aromaticum* AC homology model (wheat carbons) without substrates, products, or their analogs bound. Following nucleotide binding, E89 (E86 in the homology model, residues labeled in black) rotates in coordination with the metal. (**C**) In the *X. autotrophicus* catalyzed reactions, the γ-phosphoryl (purple) is proposed to be transferred to acetone (green) and the β-phosphoryl to bicarbonate to generate the phosphoenolacetone and carboxyphosphate intermediates, respectively, at the ATP binding site. (**D**) The two phosphorylated intermediates are hypothesized to travel 40 Å to the Mn(II) site where C–C bond formation occurs. An active site base and divalent metal catalyze the release of CO_2_, which reacts with the phosphoenolacetone to form the acetoacetate product.

The first structural data obtained for the *X. autotrophicus* AC, however, showed a molecule of AMP bound well beyond coordinating distance at approximately 40 Å from the Mn(II) (Fig. 1). An apparently structural Zn(II) is coordinated by four cysteinyl residues in the γ subunit. AMP binding was also correlated with changes in the coordination state of the Mn(II) and the opening of a water-accessible channel connecting the nucleotide and metal binding sites. These structures led us to propose a revised mechanism, consistent with the experimentally observed reaction stoichiometry in which acetone and bicarbonate are phosphorylated by the same ATP at the ATP binding site and then travel through the enzyme’s interior channel to the metal (16). Mn(II) is proposed to coordinate the phosphorylated intermediates, catalyzing C–C bond formation through a combination of inductive effects and a proton relay to the phosphate products. No experimental data describing the Mn(II)-catalyzed portion of the reaction are yet available.

AC from *Aromatoleum aromaticum* (a β-protetobacterium) has also been purified and reported with ~2.0 mole equivalents Fe, 1 Zn, and no Mn (18) in contrast to the reported metal content of *X. autotrophicus* and *R. capsulatus* discussed above. This came as somewhat of a surprise since the *A. aromaticum* AC shares >68% sequence identity with the *X. autotrophicus* AC. Given that the proposed role of the catalytic metal in AC can potentially be served by either Mn(II) or Fe(II), it is most surprising that there are ACs that use Mn in light of the lower thermodynamic affinity predicted by the IrvingWilliams series.

Here, we heterologously expressed the *A. aromaticum* and *X. autotrophicus* ACs in *Escherichia coli* under the same conditions, characterized the susceptibility of each enzyme to metal chelators, and examined metal (Mn or Fe) preference in reconstitution experiments. We observed distinct, intrinsic preferences for Mn(II) or Fe(II) for each enzyme, and correlated metal binding at the active site with specific EPR and Mössbauer spectroscopic signatures. The results suggest both an intrinsic binding preference and a specific dependence of activity in each enzyme on the correct metal.

## MATERIALS AND METHODS

### Expression and purification of recombinant ACs

AC from *X. autotrophicus* and *A. aromaticum* were expressed under the same conditions using pET-Duet system vectors (EMD Millipore, Merck, Darmstadt, Germany) in *E. coli* BL21(DE3) cells grown in lysogeny broth medium supplemented with 50 µM MnCl_2_. Both enzymes were purified as previously described (16) with slight modification. The *E. coli* cells were lysed by sonication in a 25 mM Tris, pH 7.6, 0.1 mM ethylenediaminetetraacetic acid (EDTA), and 20% glycerol buffer. The lysate was applied to a Ni(II)-NTA (nitrilotriacetic acid) column (Bio-Rad), for purification via an N-terminal 6-histidine tag on the β subunit, and eluted with a linear gradient with an imidazole buffer (25 mM Tris, pH 7.6, 0.1 mM EDTA, 20% glycerol, and 400 mM imidazole) (16). Fractions containing AC were pooled and buffer exchanged into a 20 mM Tris, pH 7.6 buffer and applied to a Q-sepharose (Bio-Rad) column. The protein was eluted with a linear gradient with a NaCl buffer (20 mM Tris, pH 7.6, 800 mM NaCl). Fractions were pooled and exchanged into a 50 mM Tris, pH 7.6, 150 mM NaCl, 10% glycerol storage buffer and stored at −80°C. Concentrations were determined by Bradford assay (25).

### Metal removal and replacement

Glassware was depleted of metals by bathing in 20% nitric acid. Plasticware was soaked in a 100 mM EDTA pH 8 bath overnight or longer. Buffers were demetalated by passage over a Chelex-100 (Sigma) column to remove metals. ACs were diluted to 10 mg/mL and dialyzed against a metal-free 50 mM HEPES, pH 7.6, 150 mM NaCl, 1 mM AMP, 10 mM EDTA, and 10 mM triethanolamine (TEA) buffer for 48 h at 4°C (12–14 kDa MWCO tubing). Dialyzed proteins were exchanged into metal-free 50 mM HEPES pH 7.6, 150 mM NaCl with or without 1 mM AMP. For reconstitution, 1 mM MnCl_2_ or 200 μM FeCl_2_ (on ice in an anaerobic Coy chamber) was added directly to AC, and the protein was incubated with metal for 1 h at 4°C and exchanged into the metal-free buffer.

### Activity

Acetoacetate production was monitored over time via a coupled assay with β-hydroxybutyrate dehydrogenase (β-HBDH) that consumes NADH (19). In a rubber-stoppered UV-vis quartz cuvette, the reaction components were added to a final concentration of 100 mM Tris, pH 7.6, 80 mM KCl, 54 mM KHCO_3_, 1 mM MgCl_2_, 343 μM NADH, 2 mM acetone, 0.25 mg/mL AC and 0.05 mg/mL β-HBDH and incubated at 37°C for 4 min prior to initiation by the addition of 2 mM ATP. NADH oxidation was monitored by UV-vis absorbance in a Cary 6000i spectrometer (340 nm). Activity assays were carried out on ≥3 independent preparations of each AC and averaged. Errors were computed as ±1 standard deviation. The measured activity of each expressed enzyme, referenced per mg of enzyme, was reported as 100%. The activities of enzymes following metal depletion and repletion, as well as the measured errors in activity, were reported relative to this amount as percentages.

### Metal analysis via inductively coupled plasma emission mass spectrometry (ICP-MS)

Metal analysis was performed on supernatants of nitric acid digested and precipitated proteins using an Agilent Infinity II autosampler coupled to an Agilent 7800 ICP-MS. The mobile phase was 2% HNO_3_, 0.5% HCl in water with a flow rate of 1 mL/min. Signals for ^55^Mn, ^56^Fe, and ^66^Zn were monitored for 32.8 s with an integration time/mass of 0.33 s per analyte. Quantification of analytes was performed in Agilent MassHunter 4.6 (version C.01.06) against a standard curve. Data were collected on the pooled biological replicates used for activity assays.

Instrument parameters were as follows: RF power 1,550 V, RF Matching 1.00 V, nebulizer Gas 0.99 L/min, option gas 0.0%, nebulizer pump 0.30 rps, S/C temperature 2°C, makeup gas 0.00 L/min, extract 1 lens 0.0 V, extract 2 lens −195.0 V, omega bias −95 V, omega lens 8.4 V, cell entrance −40 V, cell exit −60 V, deflect 0 V, plate bias −55 V, He flow rate 4.0 mL/min, octupole bias −18 V, octupole RF 200 V, and energy discrimination 3.0 V.

### EPR spectroscopy

**Fig 2:**
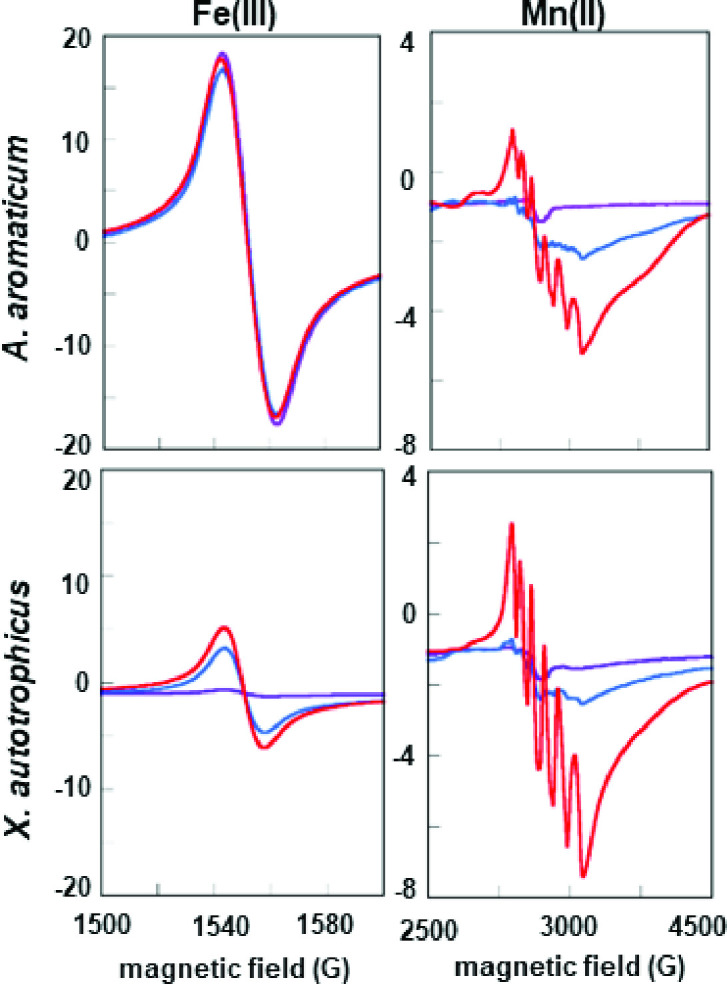
EPR spectra measured for AC from *A. aromaticum* (top) and *X. autotrophicus* (bottom) reconstituted with Mn(II). Spectra for metal-depleted enzymes are shown in purple, along with enzymes reconstituted with Mn(II) (blue) and Mn(II) plus AMP (red). Regions of the spectra corresponding to Fe(III) (left) and Mn(II) (right) are shown on different intensity (*y*-axis) scales. The 6-line spectrum is associated with Mn(II) in a 6-coordinate active site. This is proposed to form following AMP binding and repositioning of the E89 side chain in the *X. autotrophicus* AC structure (E86 in the AC from *A. aromaticum*). Protein concentrations were initially (purple traces) 73 μM (top) and 98 μM (bottom). Following reconstitution with Mn(II) (±AMP): 55 μM (top) and 48 μM (bottom). EPR conditions: 12 K, modulation amplitude 10 G, modulation frequency 100 kHz, 2 mW power, and microwave frequency 9.38 GHz. Data were measured on >3 independent preparations of each enzyme. Representative data are shown.

Samples (250 μL) were prepared in 50 mM Tris, pH 7.6, 150 mM NaCl buffer. For AMP-containing samples, 10 mM AMP and 1 mM MgCl_2_ were added. Final concentrations of the *X. autotrophicus* AC were 27 mg/mL and ~20 mg/mL, and 35 mg/mL and ~20 mg/mL for the *A. aromaticum* AC for the data reported in Fig. 2. Samples in quartz EPR tubes were frozen in liquid nitrogen. X-band EPR spectra were measured using a Bruker EMX continuous-wave spectrometer equipped with a continuous flow, liquid helium-cooled Oxford cryostat. EPR parameters were as follows: modulation amplitude 10 G, modulation frequency 100 kHz, 2 mW power, temperature 12 K, and microwave frequency 9.38 GHz. Data were measured on >3 independent preparations of each enzyme, demonstrating the reproducibility of the spectra. Representative data are shown.

### Mössbauer spectroscopy

EDTA/TEA-treated AC was reconstituted with ^57^Fe(II) in an anaerobic Coy chamber. Proteins were buffer exchanged with an oxygen- and metal-free 50 mM HEPES, pH 7.6, 150 mM NaCl, and 1 mM AMP buffer. ^57^Fe(II) was added to a final concentration of 200 μM, the protein incubated on ice for 1 h, buffer exchanged against a 50 mM HEPES, pH 7.6, 150 mM NaCl, with or without 1 mM AMP buffer, and concentrated to 500 μL and were frozen in custom polyethylene containers. The *A. aromaticum* AC samples were concentrated at 44 mg/mL (AMP present) and 79 mg/mL (AMP-free). The *X. autotrophicus* samples were concentrated at 48 mg/mL (AMP present) and 58 mg/mL (AMP-free).

Zero-field, temperature-dependent ^57^Fe Mössbauer spectra were recorded using a spectrometer operated in a constant acceleration mode. The instrument was equipped with a Janis 8DT cryostat cooled with liquid helium. Spectra recorded at 4.2 K were obtained by submerging the absorbers in liquid helium. At 80 K, we used a Cryocon 22C-120 controller and a Cernox sensor to measure the temperature of the sample which was kept in a stream of cold gas. The 14.4 keV gamma-ray source consisted of 80 mCi ^57^Co dispersed in a rhodium foil. Isomer shift values are reported against the center of a room temperature spectrum recorded for a foil of α iron. Spectral simulations were performed using the WMOSS spectral analysis software (See Co., Edina, MN) and Igor Pro codes developed in-house.

### Homology modeling

A homology model for *A. aromaticum* AC was constructed using Robetta comparative modeling (26, 27). The three Mn- and Zn-bound *X. autotrophicus* AC crystal structures (PDB ID: 5SVC for ligand-free; 5SVB for Mg^2+^-AMP bound; 5M45 for acetate and Mg^2+^-AMP bound) were used as templates. Fe(II) was inserted in the active site, and the structure was refined by force-field-based geometry optimizations. The Amber14 force field was used for the protein. The active site was parametrized from cluster models, which include the Fe center and its ligands. The N-terminus was modeled with an acetyl group and the C-terminus with an amide group. An analogous structural model was built for the Zn binding site. From these models, atomic point charges were obtained from the standard RESP procedure using electrostatic potentials obtained from density functional theory (DFT) geometry optimization using the B3LYP (28–31) exchange and correlation functional and the def2-TZVP basis set (32). These models were also used to extract equilibrium geometrical parameters. The AMP parameters were adapted from the R.E.D.D. project database (http://upjv.q4md-forcefieldtools.org). DFT calculations were performed with NWChem6.8 (33). Force field optimizations were performed with Gromacs2022 (34, 35).

## RESULTS

The ACs from *A. aromaticum* and *X. autotrophicus* were expressed in *E. coli* and their metal contents were measured by ICP-MS. The absence of known non-specific or AC-specific chaperones implies that metal incorporation is controlled only by the affinity of the enzyme for either metal in the host cytoplasm. The AC from *X. autotrophicus* was isolated with roughly the same amount of Fe and Mn bound (Table 1), in spite of having an expected twofold or greater excess of Fe in the cytoplasm of *E. coli* cultivated in LB medium, no known Mn(II) chaperones (36), and a thermodynamic bias toward Fe(II) binding predicted by Irving and Williams (15). The heterologously expressed *X. autotrophicus* had comparable Fe content and about twofold lower Mn content than previously reported for the enzyme isolated from the native host (19). It was further observed that the Mn incorporation is dependent on media composition in the native host culture when more defined mineral salt media were used (23). For heterologous expression, we are somewhat constrained in terms of media composition given that rich media is important for maximal protein expression. In contrast to what is observed for *X. autotrophicus* AC, the metal complement of *A. aromaticum* AC is almost entirely Fe with an order of magnitude lower detected Mn. As in the case of *X. autotrophicus* AC, we observed about twofold lower Fe in *A. aromaticum* AC in our heterologously expressed samples than what was observed for *A. aromaticum* AC isolated from the native host (18). Again, the somewhat lower yield could relate to suboptimal expression conditions in comparison to the defined medium used to culture the native host. Irrespective of the observed difference in the magnitude of metal loading in the two ACs the data are unambiguous in confirming that the two ACs bind metals differently, that *A. aromaticum* AC has a very strong preference for binding Fe, and that the differences in metal content of the *X. autotrophicus* and *A. aromaticum* ACs cannot be attributed to growth conditions, expression conditions, or any factors inherent to the host physiology. In addition, the *A. aromaticum* AC exhibited a greater than twofold higher specific activity than was observed for *X. autotrophicus* AC as determined in the 3-hydroxybutyrate dehydrogenase coupled assay.

**TABLE 1:** Metal-binding per AC (αβγ)_2_ heterohexamer as measured by ICP-MS, correlated with acetoacetate forming activity^*a*^

Sample	Fe	Mn	Zn	[AC] (mg/mL)	Specific activity	% Specific activity^*b*^
*Xa* as purified	0.7	0.6	1.2	41	150	100
*Xa* EDTA/TEA treated	0.1	0.1	0.1	9.6	110	73
*Xa* reconstituted with Mn(II)	0.4	0.5	0.1	6.0	170	110
*Xa* reconstituted with Fe(II)	1.4	0.2	0.1	6.5	120	80
*Aa* as purified	0.8	0.1	1.2	19	350	100
*Aa* EDTA/TEA treated	0.3	N/D	0.3	9.8	22	6
*Aa* reconstituted with Mn(II)	0.3	0.50	0.2	7.3	15	4
*Aa* reconstituted with Fe(II)	1.4	N/D	0.2	8.8	340	97

^*a*^ Abbreviations: *Xa*, *X. autotrophicus* AC; *Aa*, *A. aromaticum* AC; N/D, none detected.

^*b*^ Percent specific activity is equal to the amount of activity in nmol NADH min^−1^mg^−1^ (measured via the NADH dehydrogenase-coupled assay using UV/visible spectroscopy) recovered after EDTA/TEA treatment and metal reconstitution compared to untreated protein, expressed as a percentage of the initial, untreated specific activity (=100%). Numbers are averages of three measurements made on samples of independently expressed/isolated enzymes, and errors are ±1 standard deviation. The activity was determined by monitoring acetoacetate production via a coupled assay. See Materials and Methods.

It was previously reported that the Mn associated with *X. autotrophicus* AC was recalcitrant to chelation (23). We therefore investigated the susceptibility to metal chelators EDTA and TEA for *X. autotrophicus* and *A. aromaticum* ACs side-by-side. In contrast to what was observed previously, we were able to remove a large fraction of metals (Fe, Mn, and Zn) from *X. autotrophicus* AC (Table 1). Interestingly, the loss of metals resulted in a loss of only about 25% of the activity (Table 1). The simplest explanation for this is that a proportion of our enzyme preparation that had bound Mn was inactive and that the active *X. autotrophicus* AC is somewhat recalcitrant to the chelation of Mn as previously reported (23). Treating the *A. aromaticum* AC with the same chelation conditions also resulted in a large proportion of the metals being removed, however, activity was almost completely eliminated. This contrasts with what was observed for *X. autotrophicus* AC and suggests that *A. aromaticum* AC is more susceptible to chelation. For both ACs, a large proportion of the protein was lost under these conditions which we had optimized to observe chelation.

To provide further insights into metal preference and metal dependency, we attempted to reconstitute the remaining *X. autotrophicus* AC and *A. aromaticum* ACs with both Fe and Mn. Additional protein was lost in this procedure and given the manipulations, the data can only be treated qualitatively though they do reveal some key insights. In addition, although steps were taken to remove metals that are not bound, we cannot directly determine the amount of metal that is bound adventitiously somewhere other than the active site. For *X. autotrophicus* AC, reconstitution with Mn only resulted in 0.25 stoichiometric equivalents of metal per active site but restored the extent of the activity that was lost in chelation. Reconstitution of *X. autotrophicus* AC with Fe resulted in 0.7 equivalents of metal per active site but only a very small amount of activity was restored. The small amount of activity restored could have been a result of a small amount of Mn contaminating the Fe reconstitution since the Mn concentration is observed to increase twofold in this experiment. Slight Fe contamination was also observed in the *X. autotrophicus* AC reconstitution. For *A. aromaticum* AC, the reconstitution results were similar regarding incorporation, with Mn reconstitution yielding 0.25 equivalents for Mn and 0.70 equivalents for Fe. The activity data were much clearer, largely as a result of more effective chelation where the reconstitution with Mn resulted in no restoration of activity and reconstitution with Fe resulted in complete restoration of activity. Interestingly, 100% of the activity was restored without any addition of Zn, indicating Zn is not important for activity.

The results confirm the metal dependencies inferred by the two independent research groups studying the *X. autotrophicus* and *A. aromaticum* ACs isolated in their native hosts. Furthermore, the results indicate that the addition of Fe will not supplant the role of Mn in *X. autotrophicus* and Mn will not supplant the role of Fe in *A. aromaticum*.

To ensure these metal removal and reconstitution experiments were associated with at least a proportion of the metal-bound at the active site, we examined each AC for components in the Mn(II) EPR spectra that could be diagnostic of metal bound to the active site. Prior work suggested this may be the case. The first X-band EPR spectra measured for an AC (from *R. capsulatus*) showed a weak Mn(II) signal which intensified when Mg-AMP or Mg-ATP were added (36). Previously, we solved a series of crystal structures for the related *X. autotrophicus* AC (16), revealing Mn(II) coordinated by H150, H175, and D153 in the α subunit and a presumed structural Zn in the γ (Fig. 1).

Surprisingly, the AMP binding site was in the β subunit, about 40 Å from the Mn site, and clearly too far to directly induce the spectral changes observed by Mn(II) EPR. However, compared with the non-liganded AC, the AMP-bound structure featured a newly opened interior pocket connecting the nucleotide and metal binding sites. This large-scale structural change was accompanied by rotation of the side chain of E89 into bidentate coordination with Mn(II), which went from 5- (H150, H175, bidentate D89, and water, not shown) to 6-coordinate (Fig. 1) (16). The intense 6-line EPR spectrum observed for this species but not for the as-isolated enzyme is consistent with symmetric/octahedrally coordinated Mn(II) with an *I* = 5/2 nuclear spin.

The EPR spectrum measured for the expressed *X. autotrophicus* AC consisted of a weak, irregular signal centered near *g* = 2, similar to spectra measured previously for the AC from *R. capsulatus* (36). Upon the addition of MgAMP, the apparent Mn(II) signal sharpened and intensified, revealing a clearly discernable 6-line hyperfine pattern consistent with octahedrally coordinated Mn(II). An intense, isotropic signal near *g* = 4.5 was also observed, suggestive of Fe(III) (*S* = 5/2) in a symmetric average ligand environment, though no Fe was observed in the crystal structure for the *X. autotrophicus* AC. The Fe(III) signal did not change with the addition of AMP. We note that Fe(II) (d^6^) (*S* = 0 or *S* = 2) is undetectable under normal X-band EPR conditions. Hence, we would not expect EPR to report on protein-bound Fe(II).

Following dialysis against EDTA/TEA, we observed a pronounced decrease in the intensity of the *g* = 2 signal, and a more modest decrease at *g* = 4.5. These results suggested that the EPR-observable Fe(III), which was easily removed by dialysis against chelators, contributed to the total iron associated with the *X. autotrophicus* AC (Fig. 2; Table 1). Reconstituting this AC with Mn(II) in the presence of AMP resulted in an intense 6-line signal, indicative of Mn(II) bound in an octahedral environment at the active site, along with an increase in the *g* = 4.5 signal associated with the weakly bound Fe(III) (Fig. 2). A similar pattern was observed for the *A. aromaticum* AC, in which reconstitution with Mn(II) resulted in the same 6-line signal associated with active site bound Mn(II). This finding suggests that both enzymes undergo an AMP-dependent change in their coordination, and both are capable of binding Mn(II) in their active sites. A symmetric signal at *g* = 4.5 may likewise be associated with non-catalytically-active Fe(III) in the *A. aromaticum* AC. The iron fraction responsible for this signal is markedly less susceptible to removal by chelation and may account for the iron detected in this AC after dialysis (Table 1). We concluded that EPR could apparently be used to detect active-site associated Mn(II) in both ACs. An increase in this signal’s intensity correlated with the increase in enzymatic activity observed for the Mn(II)-depleted *X. autotrophicus* AC following reconstitution with Mn(II). While heterogeneity in the expressed, heterohexameric protein made it impossible to quantify the correlation between activity and active site Mn(II) occupancy on a per-metal basis, the trend was clearly and reproducibly observed.

The reciprocal relationship between activity and active site iron could not be examined using X-band EPR, as Fe(II) lacks a signal. We consequently examined whether ^57^Fe-Mössbauer spectroscopy, which is highly sensitive to iron oxidation state and coordination geometry (37), would reveal analogous AMP-dependent changes in coordination for ^57^Fe(II)-bound to AC. Mössbauer samples were prepared in a similar way to those used for EPR, except that excess dithionite was added to reduce all of the AC-associated iron to Fe(II). We hypothesized that at least two populations of Fe(II) sites would be present: one corresponding to the active site metal, and another non-catalytic iron pool more sensitive to chelation.

**Fig 3:**
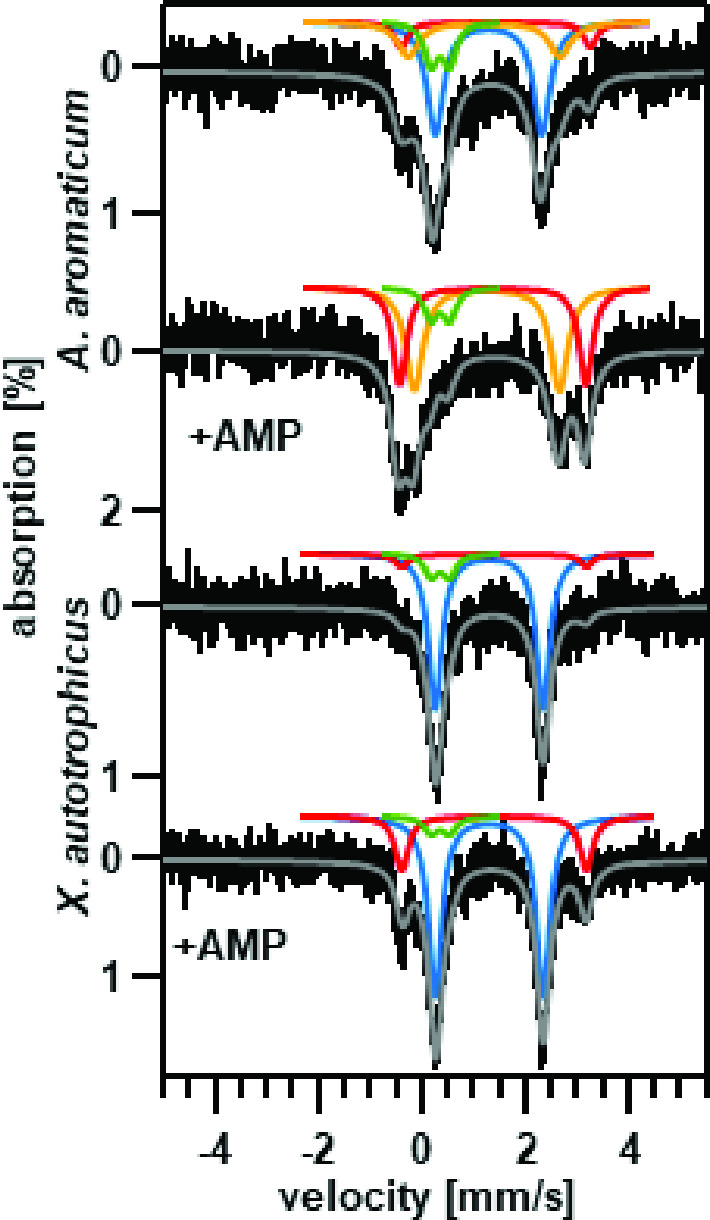
Mössbauer spectra of *A. aromaticum* AC or *X*. *autotrophicus* AC reconstituted with ^57^Fe(II). Spectra, from the top, are as follows: *A. aromaticum* AC, *A. aromaticum* AC in the presence of AMP, *X*. *autotrophicus* AC in the absence of AMP, and *X*. *autotrophicus* AC in the presence of AMP. Spectra were measured at 80 K, 0T (*A. aromaticum* AC) or 4.2 K, 0T (*X. autotrophicus* AC). Simulated Fe(II) signals are solid color lines while experimental data are shown in black. The solid gray line is the sum of all the individual spectra. The blue simulated doublet is attributed to the Fe(II) site, the red doublet to the glutamate-bound Fe(II) induced when AMP is bound, and the orange doublet to an unknown Fe(II) state. The green doublet is attributed to an instrumental artifact originating from an iron impurity in the window of our detector.

The spectrum of the expressed *A. aromaticum* AC was deconvoluted into four quadrupole doublets (Fig. 3; Table S1) three of which (blue, red, and orange) were affected by the addition of AMP and which originated from high spin (*S* = 2) Fe(II) sites. The fourth quadrupole doublet (green) was traced to an instrumental artifact, namely, to an iron impurity in the window of our gamma ray detector, and is observed for all samples investigated in this study. We hypothesize that the AMP-induced conversion of the blue doublet to the red and orange components accounts for the transition from the 5-coordinate active site (blue) to the 6-coordinate active site Fe(II) with Glu coordinated (red and/or orange). It is unclear why would there be two doublets (red and orange) for the 6-coordinate site, but it may be explained by the presence of different, but related coordination conformations. As of now, we do not have an experimentally determined structure and we are basing our hypotheses strictly on the analogies to *X. autotrophicus* AC.

The Mössbauer spectra of the *X. autotrophicus* protein exhibited two AC specific high spin (*S* = 2) Fe(II) quadrupole doublets (red and blue, Fig. 3; Table S1) which are affected by the AMP binding. In contrast to what is observed for *A. aromaticum* AC, for *X. autotrophicus* AC we observed only a small increase in the contribution of the red doublet with very little change in the blue component. The changes induced by the binding of AMP suggest that some Fe(II) is likely bound at the active site. However, the lower magnitude of the change in the red component, little or no change in the blue component, and the absence of the orange doublet observed for *A. aromaticum* AC, suggests that Fe(II) is bound differently in the two ACs such that the binding of AMP does not invoke the same changes in coordination. This is in line with the reconstitution experiments that indicate in sum that metals are bound differently in the active sites of *A. aromaticum* and *X. autotrophicus* ACs.

## DISCUSSION

Thermodynamically driven metalation should favor incorporation of Fe(II) into ACs. Yet, we have demonstrated here that two homologous carboxylases with identical first coordination spheres nonetheless exhibit distinct binding preferences for Fe(II) or Mn(II). The preference in the case of Mn(II) goes against the predicted order of the Irving–Williams series (10–12) and is even observed for the *X. autotrophicus* enzyme produced in iron-rich media and in an *E. coli* environment where Mn(II) and AC-specific chaperones are absent. The distinct binding preferences are all the more remarkable given the strict catalytic dependence of each enzyme on only its cognate metal as demonstrated here, though either Mn(II) or Fe(II) could in principle serve the proposed role as a Lewis acid in catalysis.

A preponderance of evidence has led to a set of principles for understanding metal preferences in the O_2_-activating Mn/Fe binding sites of class I ribonucleotide reductases and Mn/Fe ligand-binding oxidases (12, 38–40). These invoke biological constraints leading to metal selectivities such as pre-organized first coordination spheres that favor Mn(II) (octahedral) or Fe(II) (tetrahedral), the participation of one metal to template incorporation of the second, or specific side-chain based post-translational modifications that prohibit entry of one metal or the other. Recent work with designed metalloproteins that incorporate metals with preferences that do not follow the Irving-Williams series order do so through the participation of two metal binding sites, one of which dynamically biases the conformation of the protein to bind lower Irving-Williams metals in the second site, preferentially over Cu(II) (41, 42). The mononuclear sites in the ACs, however, lack either a pre-organized, Mn(II)-favoring ligand set or post-translational modifications. They also cannot make use of an adjacent metal site to drive metal binding cooperatively (43).

**Fig 4:**
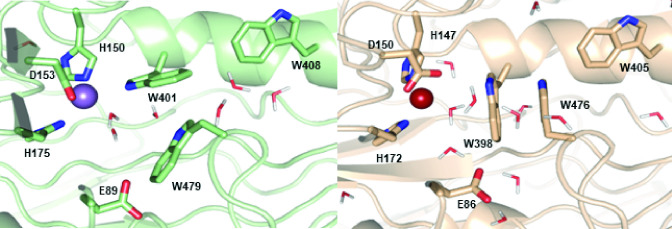
Proposed metal selectivity filter. The refined structures of *X. autotrophicus* (green) and *A. aromaticum* (tan), indicating possible differences in access to the metal (purple or rust spheres, respectively), are shown. In the *X. autotrophicus* structure, the three Trp resides (W401, W408, and W479) are arranged in a mixed stacked/T-shape that restricts solvent access to the Mn site. In the *A. aromaticum* model, however, the W398 and W479 residues are more ordered and stacked. Water molecules are shown as sticks.

Instead, metal selectivity in the mononuclear sites of the ACs must be specified outside the conserved immediate coordination environment of the metal. To gain some hypothetical understanding of how selectivity could work, we compared the available crystallographic structures for the *X. autotrophicus* AC to models of the *A. aromaticum* AC, which were constructed and energy minimized using comparative modeling of the fully solvated enzyme with the *X. autotrophicus* AC structures as a template. Comparisons of these structures highlight several differences in their outer coordination spheres, any of which could be involved in encoding metal preferences. For example, a patch of aromatic residues in the *X. autotrophicus* AC (Trp401, Trp479, Trp408, and Trp438) adopts a mixed stacked/T-shape arrangement that limits water access to the active site (Fig. 4). Such a hydrophobic obstacle course could preferentially favor passage of Mn(II) over Fe(II), because of the relative ease of exchanging solvent water for active site ligands by Mn(II). A similar patch of aromatic residues in *A. aromaticum* AC (Trp398, Trp476, Trp405) adopts a significantly more ordered stacked arrangement, granting easier water access to the active site.

Understanding the protein-based selectivity filters in these enzymes will advance our ability to emulate nature’s precise positioning of metals in complex systems. The observed selectivity also suggests a means, under the control of natural selection, by which an aerobic species (*X. autotrophicus*) could adapt an enzyme to utilize Mn(II). Such adaptation could be beneficial in the face of the decreased Fe(II) availability and the rise of harmful Fe(II)/O_2_ Fenton reactivity accompanying the oxygenation of the atmosphere.

## SUPPLEMENTAL MATERIAL

Table S1 mbio.02987-23-s0001.docx Mössbauer parameters.

ASM does not own the copyrights to Supplemental Material that may be linked to, or accessed through, an article. The authors have granted ASM a non-exclusive, world-wide license to publish the Supplemental Material files. Please contact the corresponding author directly for reuse.
