# Supplementary material for: Homologous acetone carboxylases select Fe(II) or Mn(II) as the catalytic cofactor
Source: mBio. 2023 Dec 21;15(2):e02987-23. doi: 10.1128/mbio.02987-23 (PMC10865871; doi:10.1128/mbio.02987-23)
Supplement: Table S1 — Mössbauer parameters. [file mbio.02987-23-s0001.docx]

Supplementary Information for:

**Homologous acetone carboxylases strictly select Fe(II) or Mn(II) as the catalytic cofactor**

Krista A. Shisler^1^, William M. Kincannon^2^, Jenna R. Mattice^2^, James Larson^2^, Adam Valaydon-Pillay^3^, Florence Mus^1,4^, Tamara Flusche^4^, Arnab K. Nath^2^, Sebastian A. Stoian^3^, Simone Raugei^5^, Brian Bothner^2^, Jennifer L. DuBois^2^, and John W. Peters^1,4*^

^1^Institute of Biological Chemistry, Washington State University, Pullman, Washington, USA

^2^Department of Chemistry and Biochemistry, Montana State University, Bozeman, Montana, USA.

^3^ Department of Chemistry, University of Idaho, Moscow, Idaho, USA.

^4^Department of Chemistry and Biochemistry, University of Oklahoma, Norman, Oklahoma, USA

^5^Physical Sciences Division, Pacific Northwest National Laboratory, Richland, Washington, USA.

SI.Table 1. Zero-field Mössbauer parameters used to derive the simulations shown in Figure 3. While the zero-field spectra recorded for *A. aromaticum* were obtained at 80 K, those recorded for *X. autotrophicus* were acquired at 4.2 K. The spectral component shown in green accounts for an experimental artifact that is, it originates from an iron impurity contained in the window of our gamma ray detector. The various components are assigned to: blue = Fe(II) site; red = glutamate bound Fe(II); orange = unknown Fe(II); green = instrumental artifact. The values listed in parentheses are the estimated uncertainty of the last digit.

| Sample | Site | δ  [mm/s] | ΔE_Q_  [mm/s] | Lnwdth.  [mm/s] | Area  [%] | Area  [%]  without green |
| --- | --- | --- | --- | --- | --- | --- |
| *A. aromaticum* | blue | 1.28(4) | 2.07(8) | 0.39(4) | 54(5) | 72(7) |
|  | red | 1.42(8) | 3.7(1) | 0.32(4) | 10(3) | 13(4) |
|  | orange | 1.2(2) | 2.9(2) | 0.5(1) | 11(4) | 15(5) |
|  | *green*^a^ | *0.35* | *0.35* | *0.30* | *15(4)* | *-* |
| *A. aromaticum +*AMP | red | 1.37(6) | 3.62(8) | 0.3(1) | 38(4) | 43(5) |
|  | orange | 1.25(3) | 2.81(6) | 0.4(1) | 50(4) | 57(5) |
|  | *green*^a^ | *0.35* | *0.35* | *0.30* | *12(3)* | - |
| *X. autotrophicus* | blue | 1.30(3) | 2.08(5) | 0.31(3) | 78(5) | 94(6) |
|  | red^b^ | 1.4(1) | 3.5(1) | 0.3(2) | <5(5) | <6(4) |
|  | *green*^a^ | *0.35* | *0.35* | *0.30* | *17(3)* | *-* |
| *X. autotrophicus* +AMP | blue | 1.30(3) | 2.08(6) | 0.31(3) | 72(4) | 77(5) |
|  | red | 1.40(5) | 3.55(8) | 0.31(3) | 21(5) | 23(6) |
|  | *green*^a^ | *0.35* | *0.35* | *0.30* | *7(3)* | *-* |

1. The parameters of this component were set to values determined independently of these simulations from a background spectrum obtained in the absence of a sample.
2. These parameters were derived from the analysis of the *X. autotrophicus* sample treated with AMP, see entries for the sample listed below.
